# Surface-associated lipid droplets: an intermediate site for lipid transport in human adipocytes?

**DOI:** 10.1080/21623945.2020.1838684

**Published:** 2020-10-27

**Authors:** Björn Morén, Claes Fryklund, Karin Stenkula

**Affiliations:** Department of Experimental Medical Science, Lund University, Lund, Sweden

**Keywords:** Lipid droplet, adipocytes, mitochondria, perilipin, triglycerides, lipolysis

## Abstract

Adipose tissue plays a major role in regulating whole-body energy metabolism. While the biochemical processes regulating storage and release of excess energy are well known, the temporal organization of these events is much less defined. In this study, we have characterized the presence of small surface-associated lipid droplets, distinct from the central droplet, in primary human adipocytes. Based on microscopy analyses, we illustrate the distribution of mitochondria, endoplasmic reticulum and lysosomes in the vicinity of these specialized lipid droplets. Ultrastructure analysis confirmed the presence of small droplets in intact adipose tissue. Further, CIDEC, known to bind and regulate lipid droplet expansion, clearly localized at these lipid droplets. Neither acute or prolonged stimulation with insulin or isoprenaline, or pharmacologic intervention to suppress lipid flux, affected the presence of these lipid droplets. Still, phosphorylated perilipin and hormone-sensitive lipase accumulated at these droplets following adrenergic stimuli, which supports metabolic activity at these locations. Altogether, we propose these lipid droplet clusters represent an intermediate site involved in lipid transport in primary adipocytes.

## Background

Adipose tissue holds the body’s major energy reservoir, where excess energy is stored as triglycerides in the adipocytes. In times of plenty, the lipid droplet rapidly grows in size, whereas lipase activity liberates fatty acids to support an increased energy demand in other tissues; both processes are tightly regulated by insulin. The size of the lipid droplet, which directly affects adipose cell size, is known to correlate with both cellular and systemic insulin sensitivity. The presence of enlarged, hypertrophic adipocytes is a key characteristic of impaired adipose tissue function [1], and increased adipocyte size correlates with insulin resistance and type 2 diabetes [2].

Even though the biochemical steps of triglyceride synthesis and hydrolysis are well characterized, the specific subcellular sites for esterification and release of fatty acids are less described, as previously reviewed [3]. Triglyceride synthesis and lipid droplet formation are believed to occur initially at the endoplasmic reticulum (ER) membrane until a certain size is reached and the droplet is released [4]. Uptake of external fatty acids and triglyceride synthesis has also been reported to occur at caveolae [5], which constitute plasma membrane-specific domains abundantly expressed in adipocytes, muscle and endothelial cells. Lipid droplets are encircled by a monolayer of phospholipids and cholesterol to support stability [6,7]. The amount of cholesterol correlates positively with cell size, which supports an increased need for cholesterol as the cell grows larger [8]. The droplet surface is decorated with protein involved in lipid metabolism, where the perilipins belong to the most well-characterized family [6]. Reports suggest isolated lipid droplets hold a modified ER-like membrane composition, including the ER-resident proteins BiP and calnexin [9]. Also, diglyceride acyltransferase (DGAT) connected to the lipid droplet supports both triglyceride synthesis and droplet formation [10]. During adipocyte differentiation, small lipid droplets are formed and subsequently fused into larger droplets over a period of several weeks, until mainly one large central droplet remains [11]. Thus, the fusion process is a slow, directed event as shown by long-term label-free microscopy [12], mediated via the CIDE-family of proteins, possibly via pore formation [13].

While the lipid droplet research field has been active for decades, the exact mechanisms of lipid droplet biogenesis, their spatial organization, function in cellular processes, and contribution to disease are not resolved [14]. Most studies reiterate the belief that primary, mature adipocytes contain one large lipid droplet, most likely due to histology analysis of intact adipose tissue with limited optical resolution. In contrast to a large, central lipid droplet, adipocyte cell lines fully differentiated *in vitro* exhibit multiple lipid droplets within a broad size range [15]. In recent studies, we identified lipid droplet clusters in primary human adipocytes, dispersed close to the cell surface [16,17]. Adrenergic stimulation caused an accumulation of phosphorylated perilipin (pS522) at these clusters, supporting lipolytic activity to occur at these droplets [17]. To our knowledge, only few reports have previously reported a similar lipid droplet phenotype in primary adipocytes. Lipid droplet patches were suggested to resemble sites of lipolysis [18], and small lipid droplets, decorated with mitochondria, was proposed to form following Rosiglitazone treatment [19,20]. Further, tiny microlipid droplets (<1 μm in diameter) were formed close to the cell surface following acute adrenergic stimulation [21]. With respect to the limited number of studies addressing lipid droplet organization in primary adipocytes, we have herein further characterized the presence of surface-associated lipid droplets in human adipocytes.

## Methods

### Material

CIDEC-GFP was generously provided by professor Peng Li (Fudan University, China), BFP-Sec61β was a gift from Gia Voeltz (Addgene plasmid #49,154). Acipimox (A7856), Triascin C (T4540) and antibodies towards CIDEC (HPA020553) were from Sigma. LAMP1 (ab25630), and EHD2 (154,784) from abcam, perilipin-1 pS522 from Vala Sciences, LC3B (2775S), hormone-sensitive lipase (HSL) pS563 (4139S), Caveolin-1 (3267), Rab5 (46,449), Rab11 (5589), and Calnexin (2679) from Cell Signalling Technology. Alexa fluor goat α-rabbit 568(A11036), 647(A21245), α-mouse 568(A11031), 647(A21236) secondary antibodies, Bodipy 493/503 (D3922), Bodipy 558/568 C12 (D3835), Bodipy 665/676 (B3932), and Lysotracker Deep Red (L12492) were from Invitrogen. MitoTracker RED CMXRos (M7512) and Mitotracker Deep Red (M22426) were from ThermoFisher, 10 nm gold secondary antibodies from Electron Microscopy Sciences.

### Adipocyte isolation

Subcutaneous white adipose tissue was removed during reconstructive surgery, and primary adipocytes were isolated from the tissue using an established protocol [22]. The patients, all female, were without known diabetes (type 1 or type 2) or thyroid gland dysfunction. Isolated, primary adipocytes were suspended (20% (v/v)) in Krebs Ringer Bicarbonate HEPES (KRBH) buffer, pH 7.4, containing 200 nM adenosine and 1% (w/v) bovine serum albumin (BSA). Cells kept in culture were suspended in DMEM (4.5 g/L glucose) with penicillin/streptomycin (PEST) (100 U/ml and 100 μg/ml, respectively), 200 nM phenyl-isopropyl-adenosine (PIA), and 3.5% (w/v) BSA and cultured at 37°C in 5% CO_2._

### Adipose tissue culture

Adipose tissue obtained from human subjects or mice was minced using scissors in KRBH buffer. Suspension of small tissue pieces was washed 2 times, resuspended in DMEM (containing PEST, PIA and 3.5% BSA), and kept at 37°C in 5% CO_2_ for the indicated time period (48 h). Next, adipocytes were isolated according to the protocol as described above. Cells were stained and fixed for microscopy as described in the fluorescence microscopy section.

### Lipid metabolism assay

To monitor lipid flux, we used the fatty acid Bodipy 558/568 C12 analogue, in line with a previous protocol [23]. In short, isolated cells were incubated for 24 h in 37°C incubator, followed by 2-h treatment with Bodipy 558/568 C12, washed 3–4 times and re-suspended in fresh DMEM (supplemented with PEST, PIA, and 3.5% BSA). Cells were incubated for another 24 h and labelled with MitoTracker deep red and Bodipy 493/503 prior fixation.

### Electroporation

Adipocytes were transfected as previously described [24]. In short, isolated adipose cells were suspended (40% (v/v)) in DMEM supplemented with PEST and PIA. Cells were electroporated using a square-wave pulse (400 V, 12 ms, 1 pulse), 16 µg/ml DNA/cuvette plasmid as indicated in the figure. After electroporation, the cells were transferred into DMEM with PEST, PIA and 3.5% BSA, and cultured at 37°C in 5% CO_2_.

### Fluorescence microscopy

Imaging was performed using a Nikon A1 plus confocal microscope with a 60× Apo DIC oil immersion objective with a NA of 1.40 (Nikon Instruments) and appropriate filter sets. Images were acquired with NIS-Elements, version: 4.50.02 (Laboratory Imaging). For TIRF imaging, we used a commercial TIRF system based on a Nikon Ti-E eclipse microscope equipped with a 100× Apo TIRF DIC oil immersion objective with a NA of 1.49 (Nikon Instruments), an iXon Ultra DU-897 EMCCD camera (Andor Technology), and four main laser lines: 405 (Cube, Coherent), 488 (Melles-Griot), 561 (Sapphire, Coherent), and 640 (Cube, Coherent) with corresponding filter sets. Images were usually acquired at a generous TIRF angle to allow imaging of protein stains related to lipid droplets, such as phosphorylated perilipin-1. Isolated cells were fixed using 4% paraformaldehyde and labelled with antibodies in a buffer containing 1% BSA, 1% goat serum, and 0.05% saponin 1–2 h per labelled antibody. For neutral lipid staining, Bodipy 493/503 was used together with confocal imaging. TIRF microscopy was used to detect protein stain only. For imaging of adipocytes, we used previously described protocol [25].

### Electron microscopy

For standard trans electron microscopy, human tissue was fixed overnight using 1% paraformaldehyde and 3% glutaraldehyde. Followed by dehydration using methanol series (20, 40, 60, 80, 100%) 30 min each, de-lipidated using dichloromethane for 1 h, and then rehydrated with reverse methanol series. Methanol was diluted in KRBH without BSA. The samples were fixed using OTO (osmium-tannic acid-osmium) for 1 h each, washed in KRBH without BSA, dehydrated using ethanol series and embedded in EPON. High-pressure freezing (HPF) was carried out in cells overexpressing EHD2wt to drive the droplet cluster phenotype. Freeze-substitution was done using 2% glutaraldehyde and 0.2% uranyl acetate in acetone for 3 d. Cells were embedded in lowicryl HM20 UV-polymerized. For immunogold labelling, grids were blocked using 1% BSA and labelled 1 h primary antibody as indicated, and 1 h with 10 nm gold particle conjugated secondary.

### Quantification of droplet size

Images were acquired using 8x averaging and cells with minimal background signal coming from the central droplet was chosen and thresholded using Fiji. The threshold was set to minimize background signal but still detect as much as possible of each individual droplet. Binary watershed was used to separate droplets, and uneven and unseparated signal, including background, was removed. Droplet area was acquired using Analyse particles. Assuming circularity, the diameter was calculated using the formula for circular area: A = π×r^2^ → r = √(A/π) → d = r×2. Droplet diameter was plotted using violin plots in GraphPad prism.

### Lipogenesis

Lipogenesis was measured according to the previous method [26]. In brief, adipocytes were pretreated with Triascin C (10 µM) or vehicle (methanol) for time as indicated in the figure, re-suspended in KRBH buffer containing 200 nM adenosine, 0.55 mM glucose and 3.5% BSA (w/v). In triplicates, 800 µl of the 5%(v/v) adipocyte cell suspension was added to each tube and incubated for 3 h at 37°C with 14 µl of 22 µCi/ml tritiated D-[6-3 H]-glucose (Perkin Elmer) with or without 15 nM insulin. The assay was stopped by adding 3.5 ml of 2,5-diphenyloxazole (Sigma) and 1,4-bis(5-phenloxazol-2-yl)benzene toluene-based scintillation liquid (Sigma). A zero time-point sample was included to measure the amount of glucose in the lipid phase during extraction that was not used for lipid synthesis.

### Lipolysis

Lipolysis was determined by measuring glycerol release as described [27]. In short, cells were pre-treated with Acipimox (20 µg/ml) or vehicle (water) for time as indicated in the figure, washed in KRH medium with 1% BSA, and treated with or without isoprenaline (10 nM) for 30 min. Cell medium was subsequently removed for enzymatic determination of the glycerol content. In short, 100 µl free glycerol reagent was added to 30 µl sample. Absorbance at 540 nm was measured after incubating for ~15 minutes at room temperature. Each sample was analysed in duplicate.

### Ethics statements

The human studies were approved by the local ethical committee. The human tissue was collected after written informed consent from the patients.

## Results

### Lipid droplet organization at the cell surface

**Figure 1.**
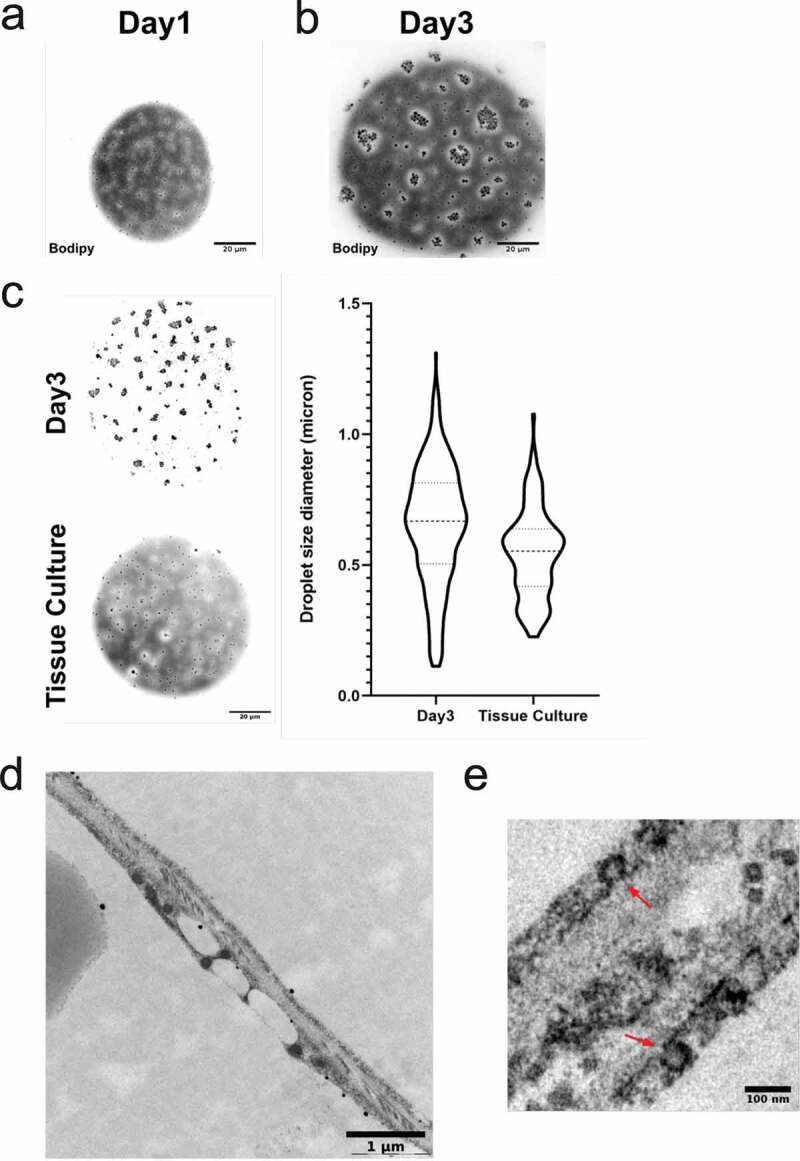
(a) Confocal image of primary human adipocytes isolated from subcutaneous adipose tissue stained with Bodipy 493/503 (neutral lipids), showing small lipid droplets dispersed at the cell surface (dispersed droplets). d1 refers to the cells being isolated and stained the same day as the tissue was excised. (b) Same as in (a) except the adipocytes were kept in culture for 2 d (corresponding to d3) before fixation and staining. The image illustrates the appearance of lipid droplet clusters. (c) Confocal image of primary human adipocytes isolated d1 and kept in culture for 2 d (corresponds to d3, upper panel) or isolated after culturing intact adipose tissue 2 d (Tissue Culture, lower panel). Quantification of surface lipid droplet sizes in both conditions are shown in violin plot to the right, illustrating the increase in size following culturing of isolated adipocytes. (ac) Scale bar = 20 µm. (d) Electron micrograph of intact human adipose tissue (d1), showing the surface droplet phenotype in one adipocyte. (e) Micrograph of the same sample as in (d) showing caveolae structures (indicated with red arrows) at the plasma membrane of two adjacent adipocytes

To monitor the distribution of lipid droplets, we applied confocal microscopy in primary human adipocytes stained for neutral lipids (Bodipy). Adipocytes that were analysed immediately after isolation (designated d1) displayed small individual lipid droplets spread equally around the cytosol (dispersed droplets) (Figure 1(a)). As expected, also the large central droplet was stained for neutral lipid. In contrast, image analysis of adipocytes kept in culture for 2 d after isolation (designated d3), displayed the formation of lipid droplet clusters close to the cell surface (Figure 1(b)). Most likely, these clusters represent expansion and aggregation of the individual dispersed droplets that were visible d1.

In order to dissect whether the culturing itself induced the cluster formation, we analysed the lipid phenotype in adipocytes that were isolated after the adipose tissue had been kept in culture ~2 d (designated Tissue Culture). These cells displayed a similar phenotype as observed in cells isolated immediately after receiving the tissue biopsy (Figure 1(c), left panel). Image analysis of both phenotypes revealed a wide range of lipid droplet sizes independent of how they were organized, even though there was a shift towards larger sizes in droplet clusters compared to dispersed droplets (average size 0.8 µm compared to 0.6 µm in diameter) (Figure 1(c), right panel). By electron microscopy, we could confirm the presence of small lipid droplets in intact adipose tissue (Figure 1(d)), and thus they are likely not a result of the adipocyte isolation procedure itself. Ultrastructure analysis also confirmed the presence of abundant caveolae domains, which verify intact adipocyte integrity (Figure 1(e)).

### Lipid droplets are decorated with CIDEC protein

**Figure 2.**
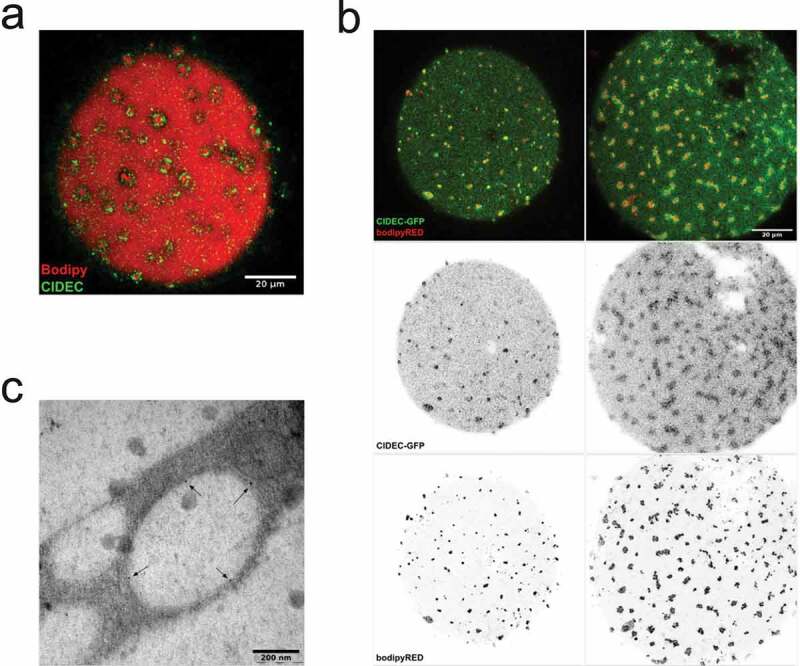
(a) Confocal image of an adipocyte stained with antibody against CIDEC (green) and Bodipy 493/503 (shown in red). (b) Confocal images of cells over-expressing CIDEC-GFP (green) stained with Bodipy colour-shifted into far red (Bodipy 665/676) (red). (ab) Scale bar = 20 µm. (c) Electron micrograph of cells exhibiting the droplet cluster phenotype, high-pressure frozen and labelled with immunogold against CIDEC (gold particles indicated with arrows)

CIDEC is known to localize at lipid droplet surfaces and to regulate lipid droplet expansion [13]. In order to examine if CIDEC was associated with the lipid droplets examined herein, we applied antibody labelling and CIDEC-GFP overexpression separately. We found a clear localization of native and overexpressed CIDEC encircling both dispersed and clustered lipid droplets (Figure 2(a,b)). Note, CIDEC labelling was also detected at the central droplet as well. Further, we performed immunogold labelling of CIDEC in human adipocytes that were overexpressing EHD2wt to induce lipid droplet cluster formation as described previously [17]. In line with observations made using fluorescence microscopy (Figure 2(a,b)), electron microscopy analysis demonstrated that CIDEC was distributed at the surface of small lipid droplets (Figure 2(c)). These data suggest that CIDEC plays a role in lipid transport between the small lipid droplets, and possibly the central droplet as well.

### Appearance of small lipid droplets independent of fatty acid flux

**Figure 3.**
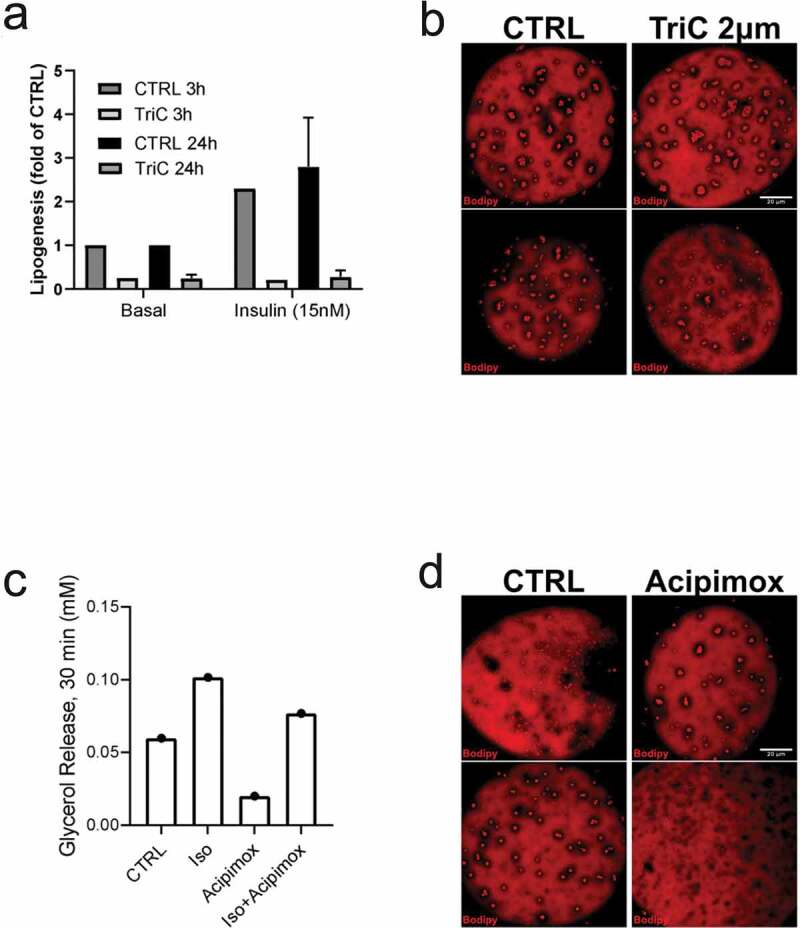
(a) Lipogenesis was determined in isolated primary human adipocytes, pre-incubated either 3 hrs or 24 hrs with Triascin C (TriC) 10 µM or vehicle (Methanol), in both non-stimulated (basal) or insulin-stimulated (15 nM) condition. N = 2 independent experiments (b) Confocal images of representative cells treated with either TriC (2 µM) or vehicle (control, CTRL) for 48 hrs followed by fixation and Bodipy 493/503 labelling. (c) Lipolysis (glycerol release) measured in isolated primary human adipocytes, pre-incubated 72 hrs with Acipimox (20 µg/ml) or vehicle (H_2_O), non-stimulated (basal) or isoprenaline-stimulated (10 nM) for 30 min. (d) Confocal images of representative cells treated with either Acipimox (20 µg/ml) or vehicle (control, CTRL) for 48 hrs followed by fixation and Bodipy 493/503 labelling. Scale bar = 20 µm

**Figure 4.**
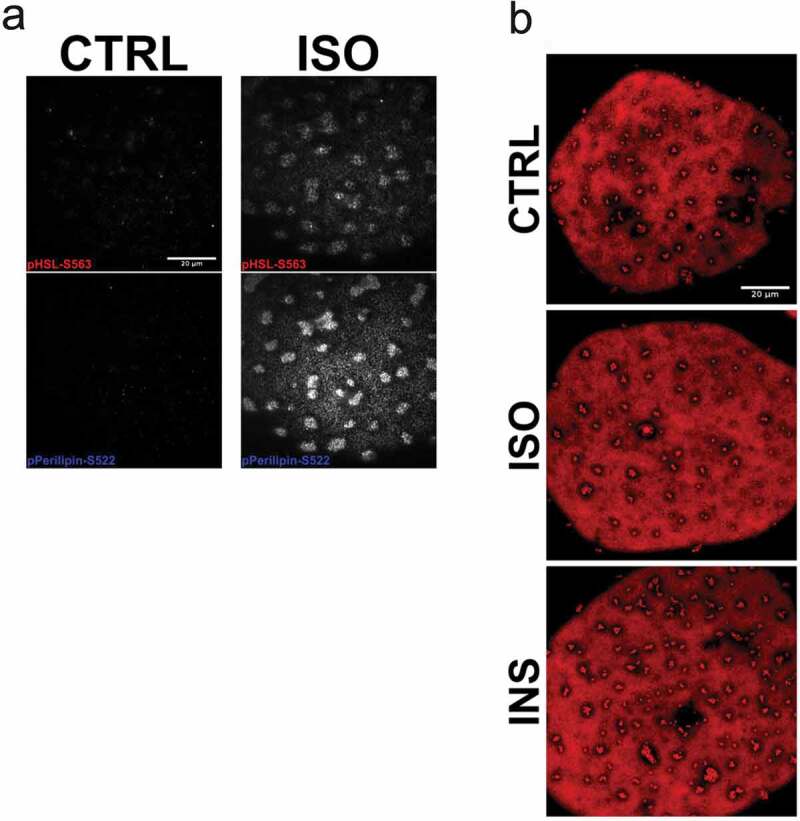
(a) TIRF images of primary adipocytes overexpressing EHD2wt, either non-stimulated (CTRL, left panel) or stimulated with isoprenaline (ISO, right panel) 100 nM for 30 min, fixed and stained with antibodies against pHSL(pS563) and perilipin-1 (pS522). (b) Long-term stimulation (24 hrs) with either isoprenaline (ISO, 10 nM) or insulin (INS 10 nM), or non-stimulated (control, CTRL). Scale bar = 20 µm

As an attempt to verify the formation of small lipid droplets is related to storage or release of fatty acids, cells were incubated with compounds known to inhibit either *de novo* triglyceride synthesis (Triacsin C) or lipolysis (Acipimox). Both acute (3 h) and prolonged (48 h) treatment with Triacsin C markedly abolished both non- and insulin-stimulated lipogenesis (Figure 3(a)) as expected. However, we could not detect any clear effect on the lipid droplet formation phenotype in TriC-treated cells compared to control (Figure 3(b)). Further, treatment with Acipimox effectively reduced basal and isoprenaline-induced lipolysis, detected as decreased glycerol release (Figure 3(c)). Still, similar to Triascin C-treatment, Acipimox did not change the lipid droplet phenotype (Figure 3(d)). In line with our previous observations [17], we found isoprenaline to induce phosphorylation of HSL (pS563) and perilipin-1 (pS522), both targets located in the vicinity of lipid droplet clusters (Figure 4(a)). This suggests these clusters are subjected to hydrolysis and thus could act as an intermediate stage of fatty acid release. However, long-term (48 h) incubation with isoprenaline or insulin did not affect the overall lipid droplet phenotype (Figure 4(b)).

### Organization of cellular organelles in the vicinity of lipid droplets

**Figure 5.**
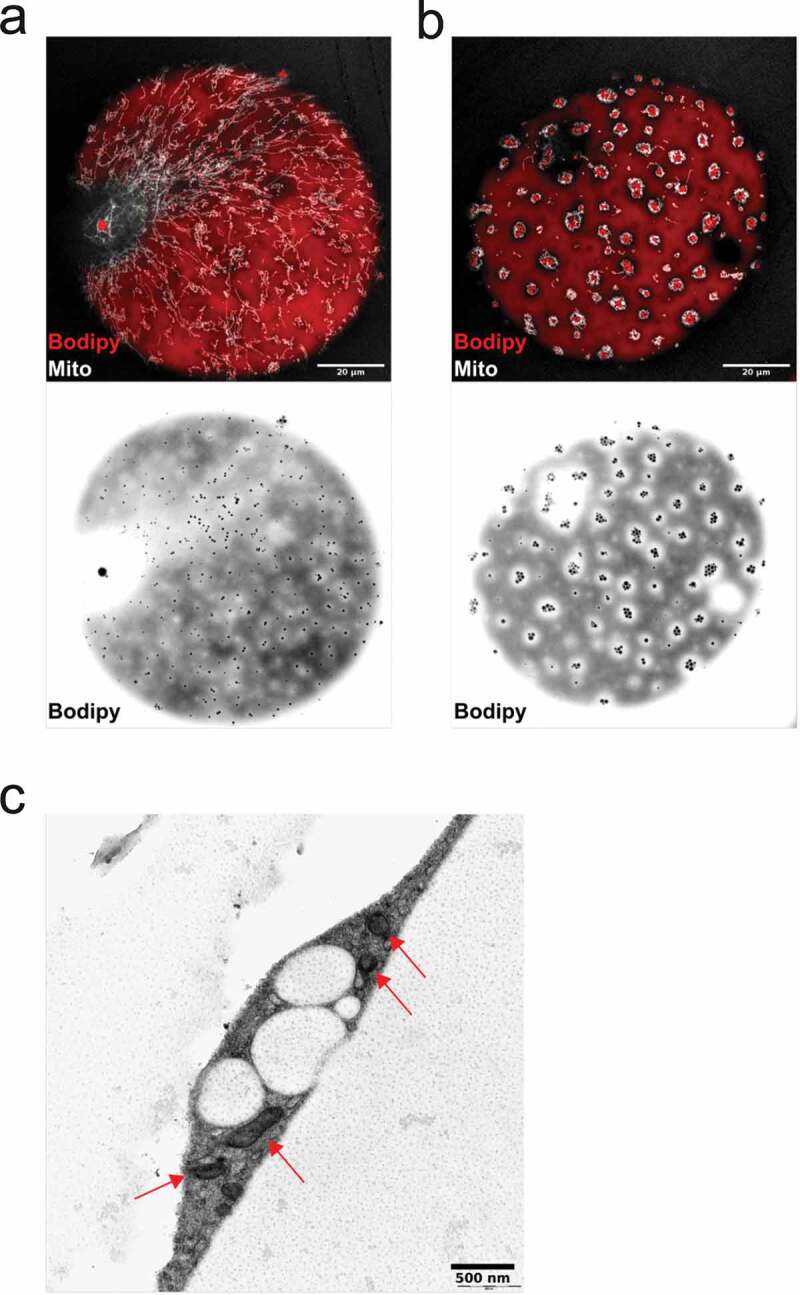
(ab) Confocal images of isolated adipocytes stained with MitoTracker (mitochondria, shown in white) and Bodipy 493/503 (neutral lipids, red). Scale bar = 20 µm. (c) Electron micrograph of high-pressure frozen cells exhibiting the droplet cluster phenotype. Mitochondria (indicated with red arrows) located in the vicinity of the droplets

Recent studies have addressed specialized mitochondria supporting triglyceride synthesis as well as fat oxidation [28]. Therefore, we set out to examine the distribution of mitochondria in relation to the surface-associated lipid droplets. In cells displaying dispersed droplets, mitochondria appeared as a highly tubular network evenly distributed throughout the cell (Figure 5(a)). Interestingly, in adipocytes that exhibited droplet clusters, mitochondria were re-distributed to profoundly locate around the droplet clusters (Figure 5(b)). Electron microscopy revealed that both dispersed droplets and droplet clusters seemed to be intrinsically connected to mitochondria which could assist in their formation/degradation (Figure 5(c)).

**Figure 6.**
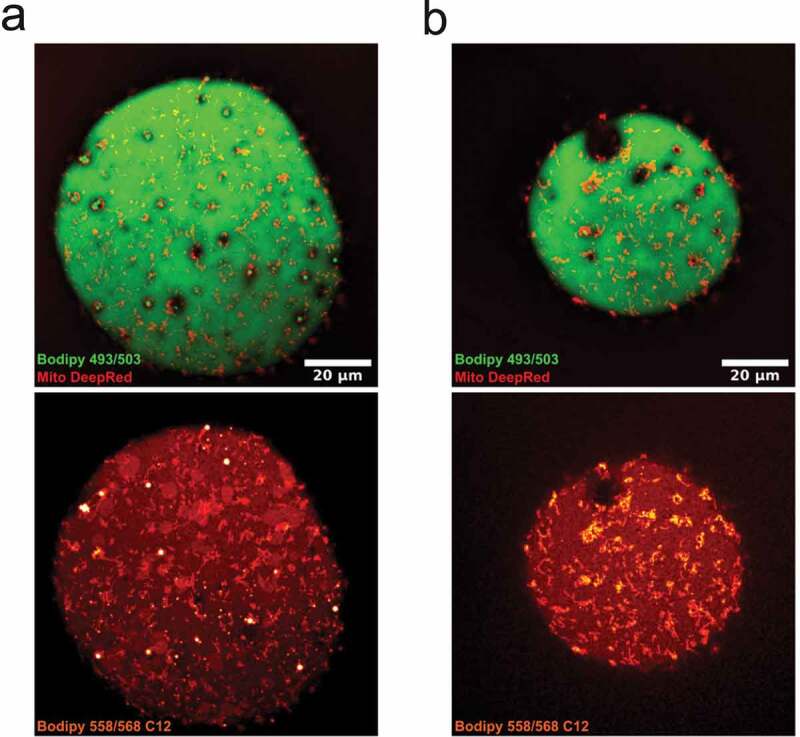
(ab) Confocal images of isolated adipocytes incubated with bodipy 558/568 C12, followed by staining with bodipy 493/503 and MitoTracker deep red. Upper panel is bodipy (green, neutral lipids) and MitoTracker (mitochondria, red), lower panel shows bodipy 558/568 C12 (red-white). Images illustrates the presence of the lipid analogue bodipy 558/568 C12 in small droplet clusters, the central lipid droplet and in mitochondria. Scale bar = 20 µm

To further explore a possible interconnection between lipid droplets and mitochondria, we used a fatty acid analogue, Bodipy 558/568 C12, as a marker for lipid metabolism [23]. In contrast to Bodipy 493/503, which stained neutral lipids in the central and surface-associated droplets, the fatty acid analogue 558/568 C12 was also, to a high extent, located in mitochondria (Figure 6(a,b)). While this observation does not dissect the direction of lipid transport, it clearly demonstrates an important role for mitochondria in lipid metabolism in primary adipocytes.

**Figure 7.**
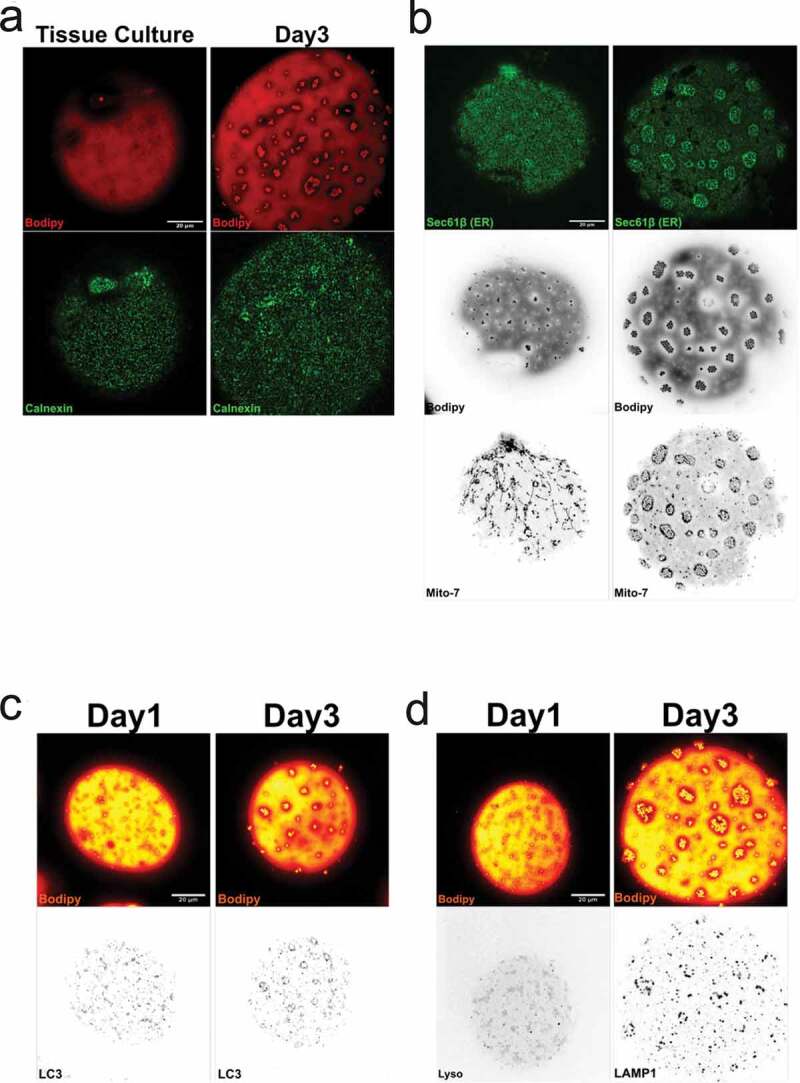
(a) Cells isolated either from tissue culture, or cells isolated and cultured for 2 d (d3) were fixed and stained for calnexin (green) and Bodipy 493/503 (red). (b) Overexpression of the ER-marker Sec61β shows structural elements all over the cell, but with an increase in signal around the clusters. Mitochondria (Mito-7) follow the same distribution pattern as in figure around the droplet clusters. (c-d) Isolated adipocytes fixed and stained with antibody against LC3, LAMP1 and bodipy 493/503 (red), either d1 (left panel) or after 2 d of culturing (d3, right panel). Scale bar = 20 µm

Further, lipid droplets are often located close to ER, the site for lipid droplet formation [4]. Here, we could confirm that ER compartments (detected by calnexin labelling and BFP-Sec61β expression) were associated to, but not exclusively, with the small lipid droplets independent of droplet phenotype (Figure 7(a,b)). While lysosomes and lipid droplets for long were considered to represent separate intracellular pathways, recent studies have addressed an interplay between these domains [29]. In a similar fashion to ER, we found lysosomes (monitored using either LAMP-1 or LC3 labelling) to be concentrated at dispersed droplets as well as droplet clusters (Figure 7(c,d)).

## Discussion

In this study, we have characterized the presence of small surface-associated lipid droplets, rarely recognized before, distinct from the large central droplet which accounts for and determines the cellular volume [30]. Based on microscopy analyses, we propose that these droplet clusters represent an intermediate stage of lipid transport in primary adipocytes. This is in agreement with previous reports, addressing a similar lipid droplet phenotype to represent sites of lipolysis [18,21]. Also, the significance of these lipid droplet compartments is supported by Lee M-J et al., which detected small lipid droplets (SLDs) in human adipocytes following Rosiglitazone-treatment [19]. In contrast to our observation, SLDs were only visible in Rosiglitazone-treated cells, whereas we find a similar phenotype independent of treatment. Possible, the discrepancy is due to the detection method since robust detection using light microscopy requires carefully optimization of pinhole and acquisition parameters, as well as immobilization of cells for higher magnification imaging [31]. Indeed, most likely, the lack of studies exploring this subgroup of lipid droplets is related to technical limitation performing image acquisition in primary adipocytes. While adrenergic stimulation was shown to generate microlipid droplets (mLD), insulin promoted lipid droplet fusion in cultured 3T3-L1 adipocytes [21]. Re-esterification of fatty acids has also been reported to increase the formation of cytosolic mLD, which was subjected to lipolytic activity [32]. In our study, using primary, human adipocytes, we observed no significant effect on lipid droplet organization following short- or long-term stimulation with either insulin or isoprenaline. We believe that these differences in lipid droplet dynamic in part are related to the distinct organization of droplets related to adipocyte models, where differentiated 3T3-L1 adipocytes hold a large number of intracellular lipid droplets of varying sizes [15] as well as tight interconnection with mitochondria, which at this stage more resembles brown adipocytes than white adipocytes [33,34]. Note, in a subset of experiments (Figure 6), we used primary adipocytes from C57Bl/6 J mice due to limited access to human tissue. Apparently, a similar droplet phenotype is present also in primary mouse adipocytes. This emphasizes the need for optimized image acquisition to detect these structures, which could explain the limited number of studies addressing this lipid domain. Even though we could not induce a change in small lipid droplet phenotype by either pharmacologic treatment or hormone stimulation, it does not rule out the metabolic activity at these sites. Rather, the fact that isoprenaline induced phosphorylation of HSL and perilipin located at droplet clusters supports the hypothesis that surface-associated lipid droplets function as an intermediate site for lipid transfer.

Further, we identified several organelles, including mitochondria, to be distributed in close vicinity of surface-associated lipid droplets. This is in line with other reports, describing a close association of mitochondria with lipid droplets [28], and lipid droplet formation to protect against mitochondrial stress [35]. In a recent review, the role of mitochondria in adipocyte biology was discussed comparing different adipocyte models and distinct distributions of cytosolic and peridroplet mitochondria and their discerning functions [36]. We also observed a change in mitochondria phenotype dependent on lipid droplet clustering, which could relate to a re-organization in metabolic flux. Using the fatty acid analogue Bodipy 558/568, we found evidence that supports fatty acids can be metabolized in mitochondria. Indeed, lipid droplets have been reported to connect to a number of organelles, including ER, mitochondria, lysosomes and peroxisomes [37]. In addition to mitochondria, we also observed an increase of lysosomes and LC3 punctae around cluster formations. The increase of proteins involved in lipid transport, autophagy as well as mitochondrial connection at these droplets indicate that they serve as centres involved in metabolism.

CIDEC is connected with lipid transport and is part of the lipid droplet coat. We found over-expressed CIDEC-GFP to be distributed on and adjacent to small droplets as a circular coat. The distribution of CIDEC suggests that lipid transport at these sites is mechanistically regulated in a similar fashion to most other cell types exhibiting small lipid droplets [13,23]. By staining (calnexin) and overexpression (Sec61β) of ER-markers we observed ER to be spread all over the cell, with some increase around droplet clusters. This suggests that, even though most observable droplets are connected to mitochondria, there also exists an intricate connection between lipid droplets and ER. Numerous articles have reported lipid droplet formation to occur in ER [4]. Since we cannot fully determine the flow of lipids, we cannot exclude that ER is relevant for the transport of lipids and lipid-related proteins. Further, we could verify some minor interaction between lipid droplets and endosomal compartments (Supplementary figure S1). However, the significance of these interactions is unclear at this point.

The fact that we observed a change in lipid droplet organization from dispersed droplets to a droplet cluster phenotype during culturing could reflect the nutritional state (starvation), loss of extracellular matrix, or deteriorating cell function. Still, the fact that we observed dispersed droplets also in intact adipose tissue supports their physiologic relevance and excludes their presence as an artefact due to cell isolation. Also, a preserved hormonal response suggests, at least partial, intact adipocyte function after cell culturing of human adipocytes. As mentioned, in most experiments, we observed lipid droplet cluster formation following cell culture. In a subset of experiments, we used overexpression of EHD2 to drive the droplet cluster phenotype as previously described [17]. In theory, this could shift the lipid flux, since EHD2 is known to affect caveolar dynamic at the plasma membrane [38], and caveolae is essential for preserved lipid transport [39]. Still, we could not observe any direct connection of caveolae bulb shapes and the smaller droplets. Instead, ultra-structure analysis demonstrated that caveolae structures were abundantly present in the membrane at other locations in intact adipose tissue. However, the hypothesis of small lipid droplets as an intermediate stage of lipid transport does not exclude a role for caveolae. For example, caveolae might still be part of the membrane in a flattened shape close to the droplet formations. Rather, the findings described herein could complement previous knowledge of caveolae and lipid transport. Since staining of EHD2 or caveolin-1 (caveolae marker), using confocal microscopy yielded a uniform membrane stain, with a certain focus around droplet clusters (Supplementary S2), we cannot exclude caveolae connection to organelles or lipid droplets, even though this is difficult to confirm at the ultrastructural level.

In summary, in this study, we provide evidence of a novel lipid droplet phenotype present in primary adipocytes, seemingly unaffected by stimulation or treatment. The presence and activation of proteins related to lipid metabolism, as well as connection with mitochondria, lead us to conclude that these droplets act as an intermediate for lipid transport in primary adipocytes.

## Supplementary Material

Supplemental Material
