# Supplementary material for: Surface-associated lipid droplets: an intermediate site for lipid transport in human adipocytes?
Source: Adipocyte. 2020 Oct 27;9(1):636–48. doi: 10.1080/21623945.2020.1838684 (PMC7595579; doi:10.1080/21623945.2020.1838684)
Supplement: Supplemental Material [file KADI_A_1838684_SM7156.zip › Supplementary Figure caption.docx]

Supplementary Figure S1. Confocal images of isolated adipocytes stained with Bodipy 493/503 (green), Mito DeepRed (red), and either Rab5 (left panel, blue) or Rab11 (right panel, blue). Scale bar=20 µm.

Supplementary Figure S2. Confocal images of isolated adipocytes stained with Bodipy 493/503 (red), and either Caveolin-1 (Cav1, lower panel, green) or EHD2 (upper panel, green). Bodipy signal only is shown in far right panel (grey). Scale bar=20 µm.
