# Supplementary figures and images for: Surface-associated lipid droplets: an intermediate site for lipid transport in human adipocytes?
Source: Adipocyte. 2020 Oct 27;9(1):636–48. doi: 10.1080/21623945.2020.1838684 (PMC7595579; doi:10.1080/21623945.2020.1838684)

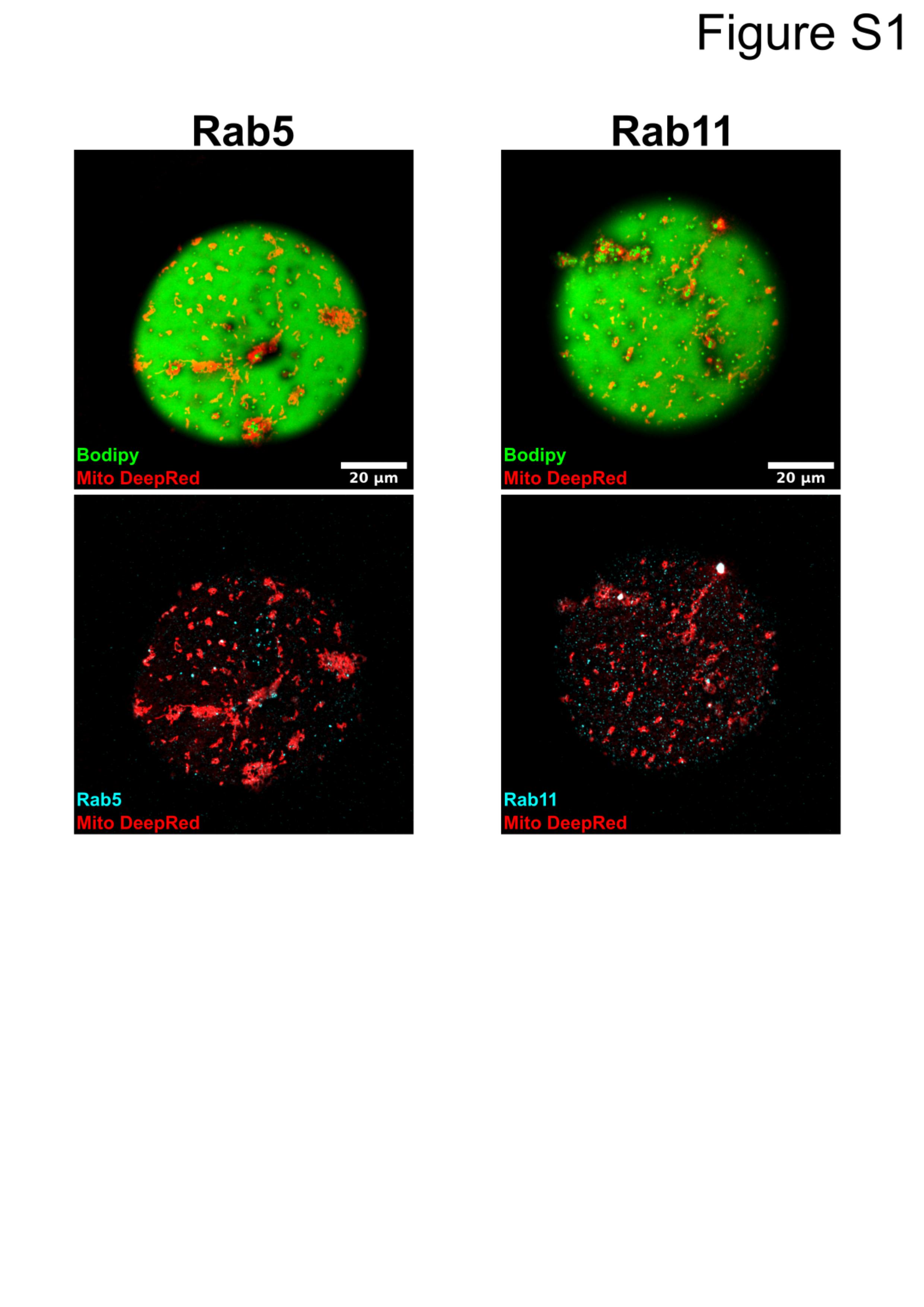

Supplement: Supplemental Material [file KADI_A_1838684_SM7156.zip › S1.jpg]

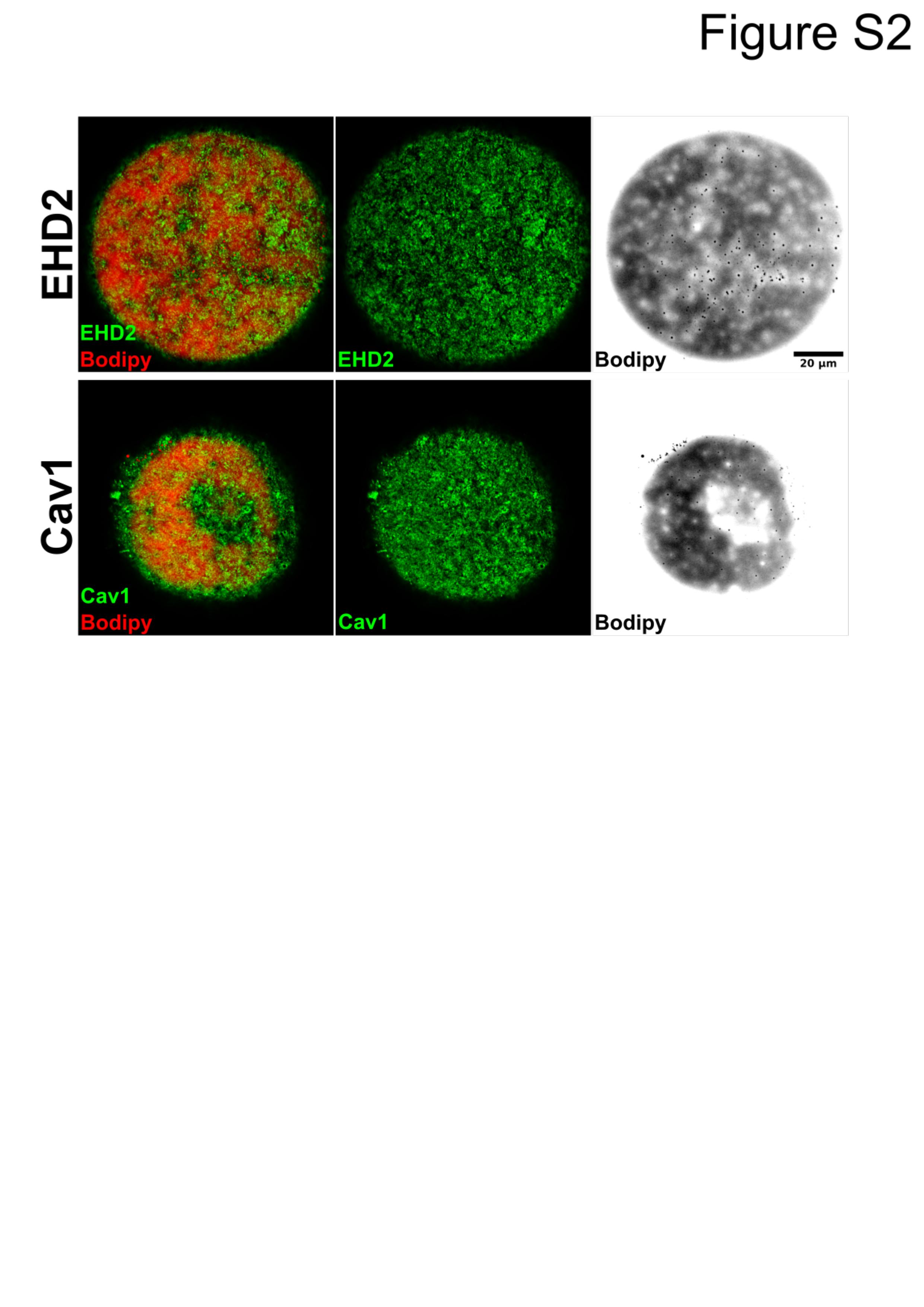

Supplement: Supplemental Material [file KADI_A_1838684_SM7156.zip › S2.jpg]
